# Necessity for detection of SARS-CoV-2 RNA in multiple types of specimens for the discharge of the patients with COVID-19

**DOI:** 10.1186/s12967-020-02580-w

**Published:** 2020-11-02

**Authors:** Yongqing Tong, Anyu Bao, Hongbing Chen, Jingtao Huang, Zhihua Lv, Lina Feng, Yun Cheng, Youna Wang, Li Bai, Wenlong Rao, Hongyun Zheng, Zegang Wu, Bin Qiao, Zhijun Zhao, Huiming Wang, Yan Li

**Affiliations:** 1 Department of Clinical Laboratory, Renmin Hospital of Wuhan University, Wuhan, 430060 China; 2 Department of Pulmonary and Critical Care Medicine of Renmin Hospital, Wuhan University, Wuhan, 430060 China; 3 Clinical Laboratory Center & Ningxia Key Laboratory of Clinical and Pathogenic Microbiology, General Hospital of Ningxia Medical University, Yinchuan, 750004 China; 4 Department of Nephrology, Renmin Hospital of Wuhan University, Wuhan, 430060 China

**Keywords:** COVID-19, SARS-CoV-2, RT-qPCR, Multiple specimens, Discharge criteria

## Abstract

**Background:**

The SARS-CoV-2 RNA was detected positive again after discharged from hospital in some COVID-19 patients, with or without clinical symptoms such as fever or dry cough.

**Methods:**

1008 severe COVID-19 patients, with SARS-CoV-2 RNA positive detected with the mixed specimen of nasopharyngeal swab and oropharyngeal swab by real-time fluorescence quantitative PCR (RT-qPCR), were selected to monitor SARS-CoV-2 RNA with the 12 types of specimens by RT-qPCR during hospitalization. All of 20 discharged cases with COVID-19 were selected to detect SARS-CoV-2 RNA in isolation period with 7 types of specimens by RT-qPCR before releasing the isolation period.

**Results:**

Of the enrolled 1008 severe patients, the nasopharyngeal swab specimens showed the highest positive rate of SARS-CoV-2 RNA (71.06%), followed by alveolar lavage fluid (66.67%), oropharyngeal swab (30.77%), sputum (28.53%), urine (16.30%), blood (12.5%), stool (12.21%), anal swab (11.22%) and corneal secretion (2.99%), and SARS-CoV-2 RNA couldn’t be detected in other types of specimen in this study. Of the 20 discharged cases during the isolation period, the positive rate of SARS-CoV-2 RNA was 30% (6/20): 2 cases were positive in sputum at the eighth and ninth day after discharge, respectively, 1 case was positive in nasopharynx swab at the sixth day after discharge, 1 case was positive in anal swab at the eighth day after discharge, and 1 case was positive in 3 specimens (nasopharynx swab, oropharynx swab and sputum) simultaneously at the fourth day after discharge, and no positive SARS-CoV-2 RNA was detected in other specimens including stool, urine and blood at the discharged patients.

**Conclusions:**

SARS-CoV-2 RNA should be detected in multiple specimens, such as nasopharynx swab, oropharynx swab, sputum, and if necessary, stool and anal swab specimens should be performed simultaneously at discharge when the patients were considered for clinical cure and before releasing the isolation period.

## Introduction

The coronavirus disease 2019 (COVID-19) has become a pandemic over the past months, with 4,962,707 confirmed cases and 326,459 deaths reported in more than 215 countries, areas, or territories by May 22, 2020 (Data from COVID-19 Dashboard website, Johns Hopkins University) [1–3]. Furthermore, there is no specific medicine or vaccine for treating with COVID-19 [4, 5], so the key measure for fighting against the epidemic is to control and reduce the source of infection.

Severe Acute Respiratory Syndrome Coronavirus 2 (SARS-CoV-2) [6], the pathogenic cause of COVID-19, has been detected in multiple types of specimen, such as nasopharyngeal swab, oropharyngeal swab, sputum, stool, anal swab, and peripheral blood, et al. [7, 8]. The patients with COVID-19 will be discharged from hospital when their symptoms meet with criteria of clinical cure, and the SARS-CoV-2 RNA is negative in two consecutive respiratory specimens by real-time fluorescence quantitative PCR (RT-qPCR) (at least 1 day of sampling time interval) [9]. However, it had been reported that the detected viral RNA results turned back to “positive” in some patients recovered from COVID-19 [10, 11]. Whether these patients were discharged with false negative results or infected again remains unclear. It is urgent to improve the criteria of viral RNA detecting for monitoring the progress of disease and discharged patients.

The aim of this study is to describe the recovery positive, which indicates that the SARS-CoV-2 RNA is not completely cleared, for the discharge of the patients with COVID-19. Firstly, we analyzed the possible sites of infection in hospitalized patients with COVID-19 by detecting viral RNA with 12 different types of specimens, including nasopharyngeal swab, oropharyngeal swab, sputum, bronchoalveolar lavage fluid (BALF), stool, anal swab, urine, peritoneal dialysis fluid (PDF), blood, sweat, cerebrospinal fluid (CSF) and corneal secretion. Furthermore, we performed the same detection with 7 different specimens, including nasopharyngeal swab, oropharynx swab, sputum, blood, stool, anal swab and urine, for clinically cured COVID-19 patients to evaluate if it is appropriate to set criteria of discharge with continuously negative results of viral RNA detection in nasopharynx swab.

## Materials and methods

### Patients

**Fig. 1:**
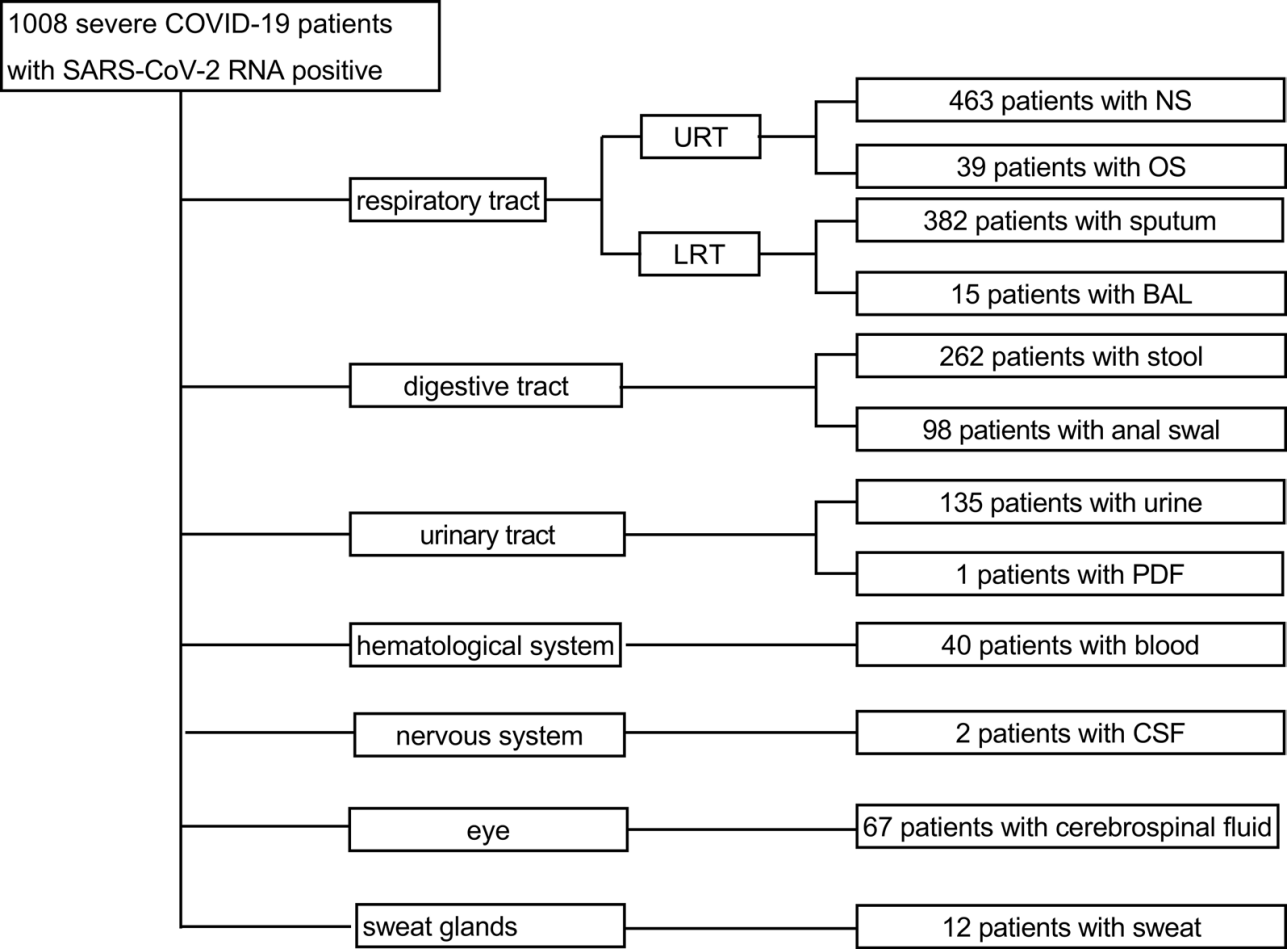
Distribution of different types of specimens in COVID-19 patients. The 12 types of specimens collected from 8 types of tissues to monitor SARS-CoV-2 from the 1008 confirmed severe patient during hospitalization. The 12 types of specimens included nasopharyngeal swab, oropharyngeal swab, sputum, BALF, stool, anal swab, urine, PDF, blood, sweat, CSF, and corneal secretion. 8 types of tissues included respiratory tract, gastrointestinal tract, urinary system, blood, eyes, the nervous system and sweat gland. NS, nasopharyngeal swab; OS, oropharyngeal swab; BALF, bronchoalveolar lavage fluid; PDF, peritoneal dialysis fluid; CSF, cerebrospinal fluid

A total of 1008 severe COVID-19 patients with positive SARS-CoV-2 RNA by RT-qPCR method with mixed specimens of nasopharyngeal swab and oropharyngeal swab, were enrolled from February 1 to February 28, 2020 hospitalized in the east branch of Renmin Hospital of Wuhan University. The diagnosis criteria of COVID-19 was in accordance with Diagnosis and treatment guidelines of coronavirus disease 2019 in China—7th Edition [12]. Severe patients should meet any of the following criteria: (1) Respiratory distress (≧30 breaths/min); (2) Oxygen saturation ≤ 93% at rest; (3) Arterial partial pressure of oxygen (PaO_2_)/fraction of inspired oxygen (FiO_2_)≦ 300 mmHg (l mmHg = 0.133 kPa). In high-altitude areas (at an altitude of over 1000 meters above the sea level), PaO_2_/FiO_2_ shall be corrected by the following formula: PaO_2_/FiO_2_ × [Atmospheric pressure (mmHg)/760]. Cases with chest imaging that showed obvious lesion progression within 24–48 h > 50%; (4) Respiratory failure and requiring mechanical ventilation; (5) Shock; (6) With other organ failure that requires ICU care. According to the patients’ diagnosis and symptoms, SARS-CoV-2 RNA was detected in the collected the specimens available from the upper respiratory tract (nasopharynx and oropharynx) and lower respiratory tract (sputum and alveolar lavage fluid), digestive system (intestinal tract and anus), urinary system (urine), blood system (peripheral blood), conjunctiva (conjunctival secretion), the nervous system (cerebrospinal fluid, CSF), peritoneal dialysis fluid (PDF), corneal secretion and sweat glands (sweat) for each hospitalized patients with COVID-19 (Fig. 1). All of the specimens were collected from the first day to end day during the hospitalization in the severe patients. This study was approved by the ethics committees of the Renmin Hospital of Wuhan University (WDRY2020-K078) and was exempted from the need for informed consent.

The 20 discharged cases of COVID-19, the criteria [12] for which was the SARS-CoV-2 virus RNA detection negative in two consecutive respiratory specimens (at least 1 day of time interval of sampling) for patients who have reached the standards of isolation period (14 days) after clinical cured, during the isolation period were selected to detect SARS-CoV-2 RNA with multiple specimens including nasopharyngeal swab, oropharyngeal swab, sputum, stool, anal swab, urine and blood. This study was approved by the ethics committees of the Renmin Hospital of Wuhan University (WDRY2020-K078) and written inform consent was obtained from the patients.

### Specimen collection and pre-processing

All collected specimens were pre-processed before RNA extraction. The PBMCs (peripheral blood mononuclear cells) were isolated from peripheral blood specimens with lymphocyte separation buffer. The urinary specimens were centrifuged at 3500 rpm for 20 min to get the sediment. Sputum was liquefied with 4% NaOH for 5 min. The bean-sized stool specimens were mixed with saline solution for blending. All swab specimens were mixed with cell storage buffer and vortexed for 5 to 10 s.

### RT-qPCR to detect the SARS-CoV-2 RNA

RNA in the specimens were extracted using Magnetic Beads RNA Extraction Kit (Health Gene Technologies, Ningbo, China) and SuperPure automatic nucleic acid extraction instrument (Fosun Pharma, Shanghai, China) according to manufacturer’s protocol. SARS-CoV-2 RT-qPCR Kit (Shanghai Huirui Biotechnology, Shanghai, China) and Roche Light Cycler 480 (Roche, Basel, Switzerland) were used to detect the expression of SARS-CoV-2 ORF1ab gene, N gene and internal label gene. Specimen with a Ct value less than 40 was considered to be positive. In this study, three negative control, one positive control and one weakly positive were randomly placed in specimens to detect at the same time.

### Statistical analysis

Descriptive analysis of the variables were expressed as number (%). The measurement data that meet the normal distribution were expressed as Mean ± standard deviation (Mean ± SD).

## Results

### Detection of SARS-CoV-2 RNA in 12 types of specimens of hospitalized COVID-19 patients

SARS-CoV-2 virus may attack different tissues and organs of human body. In order to understand the distribution of SARS-CoV-2 RNA in different specimens, we collected 1516 available specimens from 1008 severe COVID-19 patients including 463 (30.54%) cases of nasopharyngeal swab, 39(2.57%) cases of oropharynx swab, 382 (25.20%) cases sputum, 15 (0.99%) cases of BALF, 262 (17.28%) cases of stool, 98 (6.46%) cases of anal swab, 135 (8.91%) cases of urine, 1 (0.07%) case of peritoneum fluid, 40 (2.64%)cases of blood, 2 (0.13%) cases of cerebrospinal fluid, 67(4.42%) cases of corneal secretion and 12 (0.79%) cases of sweat (Fig. 1).

**Fig. 2:**
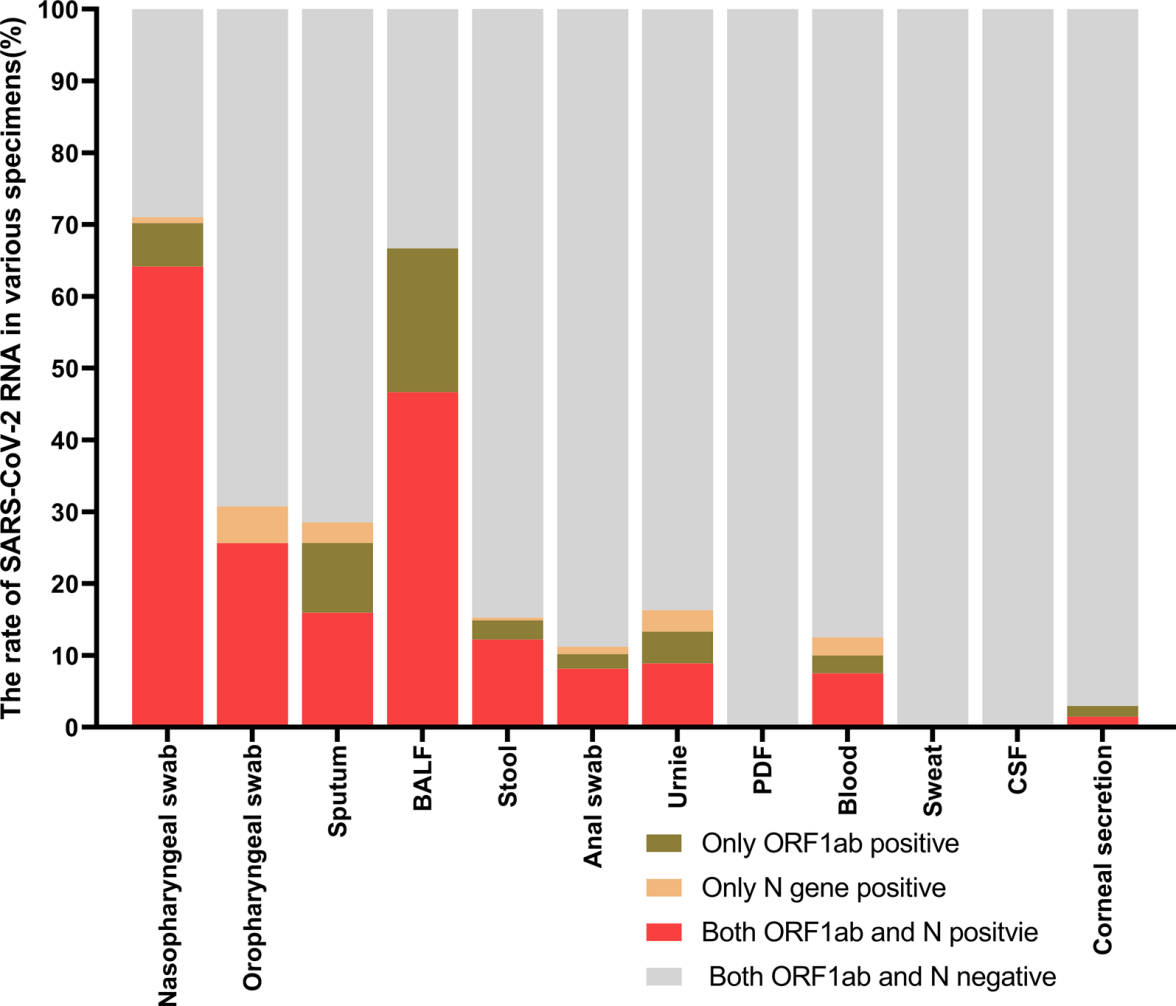
Positive rate of SARS-CoV-2 RNA detected in different specimens from COVID-19 patients. It was defined as positive when the ORF1ab gene and N gene were both positive at the same time. It was defined as suspicious when ORF1ab gene or N gene was positive, which should be resampled to detect again after 24 h

We performed SARS-CoV-2 RNA detection with the 12 types of specimens from 1008 hospitalized patients with COVID-19 (Table 1). The double positive for both ORF1ab gene and N gene were 64.15% (297/463) in nasopharyngeal swab, 46.67% (7/15) in BALF, 25.64% (10/39) in oropharynx swab, 15.97% (61/382) in sputum, 12.21% (32/262) in stool, 8.89%(12/135) in urine, 8.16% (8/98) in anal swab, 7.50% (3/40) in blood and 1.49% (1/67)in corneal secretion (Fig. 2). The single positive for either ORF1ab gene or N gene were 20.00% (3/15) in BALF, 12.57% (48/382) in sputum, 7.40% (10/135) in urine, 6.91% (32/463) in nasopharyngeal swab, 5.13% (2/39) in oropharynx swab, 5.00% (2/40) in blood and 3.05% (8/262) in stool (Fig. 2). No SARS-CoV-2 RNA was detected in PDF and CSF specimens.

**Table 1:** SARS-CoV-2 RNA in different specimens from COVID-19 patients

Types of specimen	Sample number	ORF1ab and N genes both positive		Only ORF1ab gene positive		Only N gene positive		ORF1ab and N genes both negative	
		No.	Rate (%)	No.	Rate (%)	No.	Rate (%)	No.	Rate (%)
Nasopharyngeal swab	463	297	64.15	28	6.05	4	0.86	134	28.94
Oropharyngeal swab	39	10	25.64	0	0.00	2	5.13	27	69.23
Sputum	382	61	15.97	37	9.69	11	2.88	273	71.47
BALF	15	7	46.67	3	20.00	0	0.00	5	33.33
Stool	262	32	12.21	7	2.67	1	0.38	230	87.79
Anal swab	98	8	8.16	2	2.04	1	1.02	87	88.78
Urine	135	12	8.89	6	4.44	4	2.96	113	83.70
PDF	1	0	0	0	0	0	0	1	100
Blood	40	3	7.50	1	2.50	1	2.50	35	87.50
Sweat	12	0	0	0	0	0	0	12	100
CSF	2	0	0	0	0	0	0	2	100
Corneal secretion	67	1	1.49	1	1.49	0	0	65	97.02

*BALF* Broncho alveolar lavage fluid, *PDF* peritoneal dialysis fluid, *CSF* cerebrospinal fluid

### Detection of SARS-CoV-2 RNA in 7 types of specimens from discharged patients with COVID-19

In order to explain the cause about so-called recovered positive, we further analyzed 7 types of specimens of nasopharyngeal swab, oropharyngeal swab, sputum, stool, anal swab, urine and peripheral blood in 20 discharged patients with COVID-19 during quarantine time (14 days). The SARS-CoV-2 RNA positive was found in 8.1 ± 3.4 days after discharge, ranging from 4 days to 14 days (Table 2). All of the 6 patients showed positive viral RNA for at least one gene in nasopharyngeal swab, 2 patients had both ORF1ab and N gene positive, another 3 patients only showed ORF1ab positive and 2 patients showed N gene positive. The double positive of both ORF1ab and N gene RNA were found in 5 cases at least in one specimen simultaneously, while 2 of which in sputum, 1 of which in nasopharynx swab, 1 of which in anal swab and 1 of which in the 3 specimens (nasopharynx swab, oropharynx swab and sputum) simultaneously. No positive SARS-CoV-2 RNA was detected in the specimens of stool, urine and blood of the 20 discharge patients (Table 2).

**Table 2:** SARS-CoV-2 RNA positive in different specimens from the discharged COVID-19 patients in isolation period

Patients		Gene	Nasopharyngeal swab	Oropharyngeal swab	Sputum	Stool	Anal swab	Urine	Blood
Number	Isolated days								
P3	6	ORF1ab	+	–	–	–	–	–	–
		N	+	–	–	–	–	–	–
P6	8	ORF1ab	+	–	+	–	–	–	–
		N	–	–	+	–	–	–	–
P7	9	ORF1ab	+	–	+	–	–	–	–
		N	–	–	+	–	–	–	–
P8	4	ORF1ab	+	–	–	–	–	–	–
		N	–	+	–	–	–	–	–
P12	14	ORF1ab	–	–	–	–	+		–
		N	+	–	–	–	+	–	–
P15	4	ORF1ab	+	+	+	–	–	–	–
		N	+	+	+	–	–	–	–

+, positive; −, negative

**Fig. 3:**
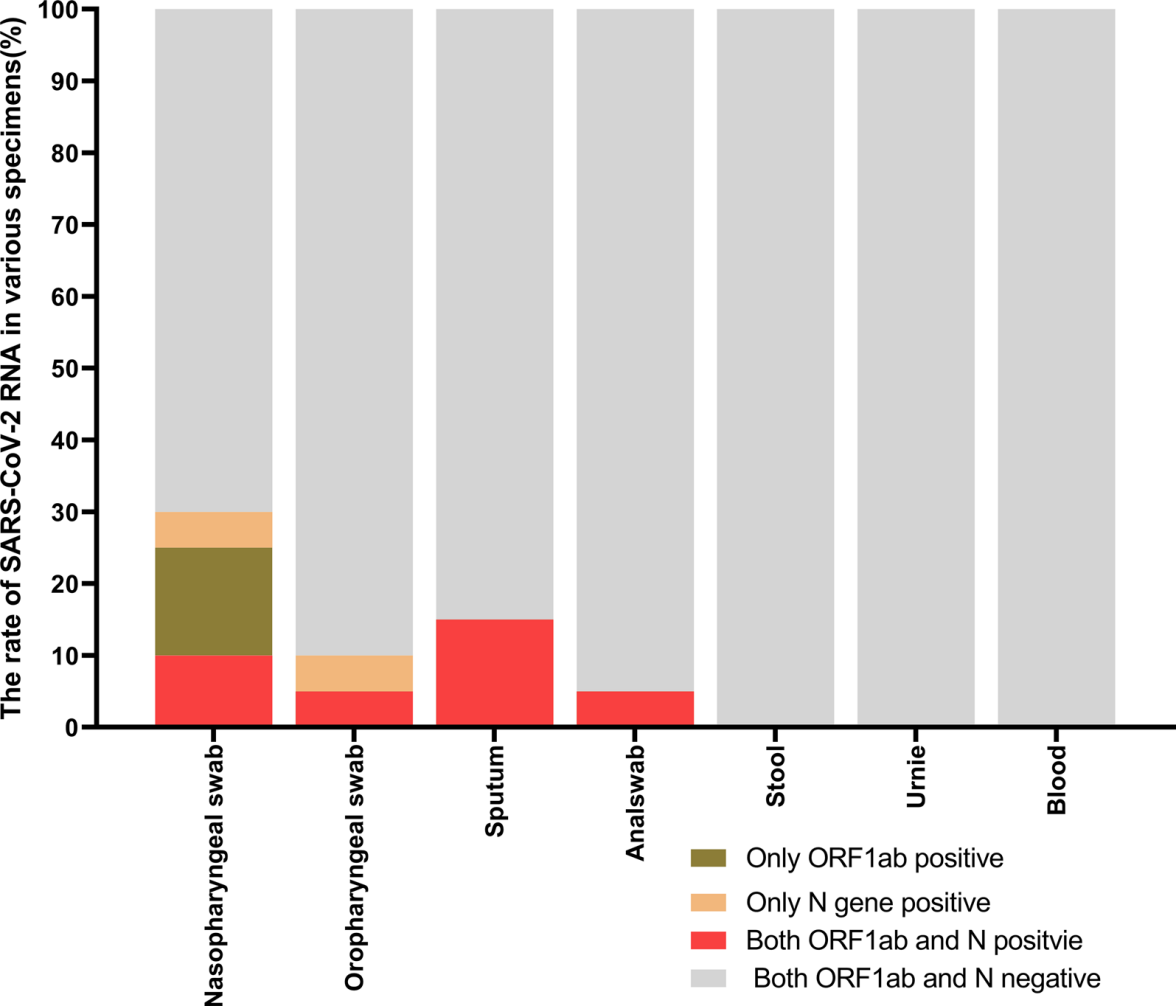
Positive rate of SARS-CoV-2 RNA detected in different specimens from the discharged COVID-19 patients in their isolation period. SARS-CoV-2 RNA was detected in 7 types of specimens before discharging. It could be considered positive when the ORF1ab and N genes were both positive at the same time. It could be considered that SARS-CoV-2 RNA was negative, and the virus was dead when only ORF1ab or N gene was positive

During the quarantine time of these 20 patients, the positive rate of SARS-CoV-2 was 30% (6/20) in nasopharyngeal swab, 10% (2/20) in oropharyngeal swab, 15% (3/20) in sputum, and 5% (1/20) in anal swab, respectively. The double positive of both ORF1ab and N gene RNA were found in 25% (5/20) cases at least in one specimen simultaneously (Fig. 3).

## Discussion

There has been increasing evidences express that SARS-CoV-2 RNA could be detected not only in respiratory tract but also in gastrointestinal tract [7, 13]. In this study, 1008 hospitalized severe COVID-19 patients were detected positive SARS-CoV-2 RNA in 12 types of specimens collected from respiratory tract, gastrointestinal tract, urinary system, blood, eyes, the nervous system and sweat. The nasopharyngeal swab specimens showed the highest positive rates (71.06%), followed by BALF (66.67%), oropharyngeal swab (30.77%), sputum (28.53%), blood (12.5%), stool (12.21%) and anal swab (11.22%). We also found the SARS-CoV-2 in urine (16.30%). These features of our results of the viral positive rate among various tissues were very different from a previous study [14]. In this study, the highest SARS-CoV-2 RNA positive rate was 71.06% in nasopharyngeal swab, followed by 66.67% in BALF, 30.77% in oropharynx swab, 28.53% in sputum, 16.30% in urine, 12.50% in blood, 12.21% in stool, 11.22% in anal swab, and 2.99% in corneal secretion. These results showed that the specimen should firstly collect nasopharyngeal swabs, followed by oropharyngeal swabs, and collect sputum, stool, anal swabs or blood. The specimen of BALF could be collected for patients with bronchial intubation in inpatient. The specimen of corneal secretion may be collected if there were eye’s symptoms for the patients with suspected COVID-19.

The SARS-CoV-2 RNA was found turning back to positive in some patients after discharge for 1 month or longer time. It is urgent to know whether the recovery positive viral RNA is caused by second infection or resulted from uncured patient itself. Therefore, our data bring to the focus of discussion whether it is appropriate to perform viral RNA detection only in two consecutive respiratory specimens (at least 1 day of time interval of sampling) for patients who have reached the standards of quarantine time (14 days) after clinical cured and discharge after treatment [9]. In this study, the SARS-CoV-2 RNA was found in respiratory tract, gastrointestinal tract, urinary system, blood and eyes, which means this virus may appear almost everywhere in human body. So we selected seven types of specimens of nasopharynx swab, oropharynx swab, sputum, stool, anal swab, urine and blood to detect SARS-CoV-2 RNA simultaneously for 20 patients who were clinically cured but still in quarantine time based on the findings of organ infection with SARS-CoV-2 and the convenience of sample collection.

Among 20 discharged patients, 5 of them expressed both ORF1ab gene and N gene RNA positive, in which 2 cases showed positive in sputum, 1 case showed positive in nasopharynx swab, 1 case showed positive in anal swab, and 1 case in 3 specimens (nasopharynx swab, oropharynx swab and sputum) showed positive simultaneously. These 5 cases were diagnosed as carriers of SARS-CoV-2. This result showed that the SARS-CoV-2 recovery positive might indicate that patient had not been fully cured when discharged at that time, though it met the criteria of discharge. The results didn’t seem to support the possibility of reinfection of the virus. As a result, the current discharge criteria could be improved according to the clinical findings and the sole detection of SARS-CoV-2 RNA in respiratory tract specimen seemed inadequate. It is necessary to collect multiple types of specimens to detect the viral RNA before the discharge of the patients, though they may meet the criteria of clinical cure. Once the patient is detected positive for SARS-CoV-2 RNA in the quarantine time, another 14 days’ isolation will be recommended until viral RNA become negative in all of 7 types of the specimens.

The 7 types of specimens that we used in this study may be a good choice, but if the patients have other concurrent diseases, it may also enlarge the scope of additional enrolled specimens to study. Moreover, a larger sample needs to be enrolled to exclude the possibility of peritoneal fluid and CSF containing the SARS-CoV-2 RNA in our cohort. In addition to the 5 cases, another 1 case (P8 at Table 2) was only detected ORF1ab gene RNA from nasopharynx swab and N gene RNA from oropharyngeal swab in the quarantine time, this patient did not meet the criteria of viral carrier. This may suggest that SARS-CoV-2 virus is dead and is being cleared by the patients. It is known that false negative of RNA detection in respiratory tract specimens is unavoidable in the severe patients [15]. Therefore, multiple types of specimen should be analyzed simultaneously to exclude the possibility of the discharge patients as the source of infection again.

## Conclusions

SARS-CoV-2 could exist in the various specimens came from different tissues and organs. It is the necessity for detection of SARS-CoV-2 RNA in multiple types of specimens at discharge of the patients with COVID-19. SARS-CoV-2 RNA should be recommended to detect before releasing the isolation period in order to avoid the discharge of patients with false negatives.

## Data Availability

All data generated or analyzed during this study are included in this published article.

## Abbreviations

COVID-19: Coronavirus disease 2019
SARS-CoV-2: Severe Acute Respiratory Syndrome Coronavirus 2
RT-qPCR: Real-time fluorescence quantitative PCR
PBMCs: Peripheral blood mononuclear cell
CSF: Cerebrospinal fluid
PDF: Peritoneal dialysis fluid
